# A Case of Bilateral Interconnected Abductor Pollicis Longus Accessory Tendon and Abductor Pollicis Brevis Accessory Head, With Absent Extensor Pollicis Brevis

**DOI:** 10.7759/cureus.108719

**Published:** 2026-05-12

**Authors:** Brittney L Tatchell, Sarah E McConnell

**Affiliations:** 1 Department of Neuroscience, University of Rochester School of Medicine and Dentistry, Rochester, USA

**Keywords:** abductor pollicis brevis, abductor pollicis longus, anatomical variation, digastric, extensor pollicis brevis

## Abstract

Anatomical variations in the abductor pollicis longus and extensor pollicis brevis muscles are frequently reported in the literature and can raise implications for functionality, inflammatory conditions, and surgical considerations. This case study presents a unique combination of morphological features affecting the abductor pollicis longus (APL), abductor pollicis brevis (APB), and extensor pollicis brevis (EPB) muscles. It also discusses the potential etiology and functional and clinical implications of this variation. Phase 1 medical students dissected the forearm and hand of a male donor during their formal anatomy curriculum, revealing a bilateral interconnection between an accessory tendon of APL and an accessory head of APB, as well as the absence of EPB. This variation likely resulted from the aberrant separation of muscle precursors and the failure of regression of a muscle slip in the hand during embryonic development. Probable functional implications include weakened thumb extension and opposition and an altered range of motion at the affected joints. Understanding the diversity of anatomical variations in the forearm and hand is important for effective treatment of related inflammatory conditions.

## Introduction

Muscle and tendon variations of the forearm and hand are commonly reported in the surgical and anatomical literature. The abductor pollicis longus (APL) is the most frequent muscle to exhibit variations affecting the thumb [1]. The APL originates from the posterior aspect of the proximal shaft of the ulna, the interosseous membrane, and the middle third of the radius [2]. It inserts distally just proximal to the wrist, often as two tendon slips to the radial side of the base of the first metacarpal and the trapezium. This attachment sometimes includes a connection to the abductor pollicis brevis (APB). The primary function of the APL is abduction of the thumb at the carpometacarpal joint. It can also aid the actions of APB and the extensor pollicis brevis (EPB). The APL is innervated by the posterior interosseous nerve (C7-C8) and receives vascular supply proximally from the posterior interosseous artery and distally from the anterior interosseous artery. Variations of the APL include tendon splitting, duplication of muscle bellies or tendons, variation in attachment location, fascial connections between muscles, muscle absence, and the presence of novel (supernumerary) muscles [1].

The APB originates from the flexor retinaculum and may have fibrous connections to the underlying scaphoid and trapezium bones [2]. It attaches distally to the base of the proximal thumb phalanx on the radial side; fibers also merge with the extensor expansion of the thumb. The primary action of the APB is abduction of the thumb at the carpometacarpal joint. It is innervated by the recurrent branch of the median nerve (C8-T1) and receives blood supply from the superficial palmar branch of the radial artery. Variations of the APB include a proximal attachment at the styloid process of the radius, muscular slips to the APL or neighboring muscles, additional muscle heads, or a complete absence of the muscle [1].

The EPB originates from the posterior shaft of the radius and interosseous membrane distal to the APL. It inserts at the base of the proximal thumb phalanx. The primary action of the EPB is to extend the thumb at the metacarpophalangeal and carpometacarpal joints. It is innervated by the posterior interosseous nerve (C7-8) and receives blood supply proximally from the posterior interosseous artery and distally from the anterior interosseous artery. The EPB is unique to humans and develops from the same precursor muscle mass as the APL [1,3]. Variations of the EPB include absence, fusion with the APL or EPL, or variable distal attachments.

Differences in muscle organization may have clinical implications, including altered muscle action and joint function that can predispose patients to inflammatory conditions and may impact the effectiveness of surgical treatment. Therefore, it is important to understand the extent of anatomical variations in this region.

The current case study details a previously undescribed combination of muscle and tendon variations affecting the APL, APB, and EPB and discusses the associated developmental biology, functional impacts, and clinical significance.

## Case presentation

Dissection

Phase 1 medical students performed a routine dissection of the forearm and hand on a 91-year-old male donor during gross anatomy laboratory sessions. The curriculum utilizes a regional approach in which students sequentially dissect the anterior forearm, followed by the palm, and finally the posterior forearm and hand. The dissection revealed identical bilateral variations of the APL and ABP with a complete absence of the EPB. Anatomy faculty conducted secondary dissection and cleaning of the forearm and hand after the conclusion of the course to confirm attachments and innervation of the anomalous muscles.

Findings

**Figure 1:**
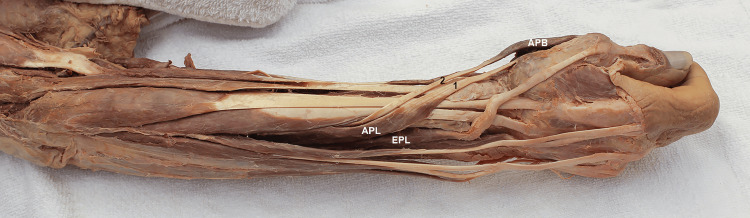
Posterolateral view of the right forearm showing two APL tendons. The first APL tendon is typically attached to the base of the first metacarpal. The second tendon is interconnected with the lateral head of the APB. The EPB muscle was absent. APL: abductor pollicis longus; 1: typical APL tendon; 2: accessory APL tendon; APB: abductor pollicis brevis; EPL: extensor pollicis longus.

**Figure 2:**
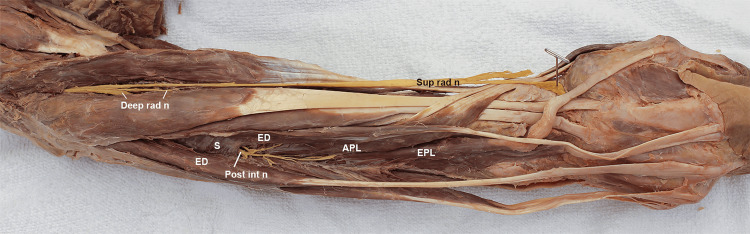
Posterior view of the right forearm demonstrating innervation. The posterior interosseous nerve emerged from beneath the supinator muscle to innervate the deep muscles of the forearm, including APL. The superficial branch of the radial nerve is stabilized distally with a pin. APL: abductor pollicis longus; deep rad n: deep branch of the radial nerve; ED: extensor digitorum (divided); EPL: extensor pollicis longus; Post int n: posterior interosseous nerve; S: supinator; Sup rad n: superficial branch of the radial nerve. Nerves are highlighted in yellow.

The APL was characterized by a single proximal muscle belly originating from the posterior radius, interosseous membrane, and ulna. The muscle terminated distally in two tendons (Figure 1). The first tendon exhibited typical attachment to the base of the first metacarpal. The second tendon was continuous with the lateral (accessory) head of the APB, creating a digastric APL-APB muscle without an intermediate bony attachment. The APL muscle belly was innervated by the posterior interosseous nerve (Figure 2) and received blood supply from the posterior interosseous artery.

**Figure 3:**
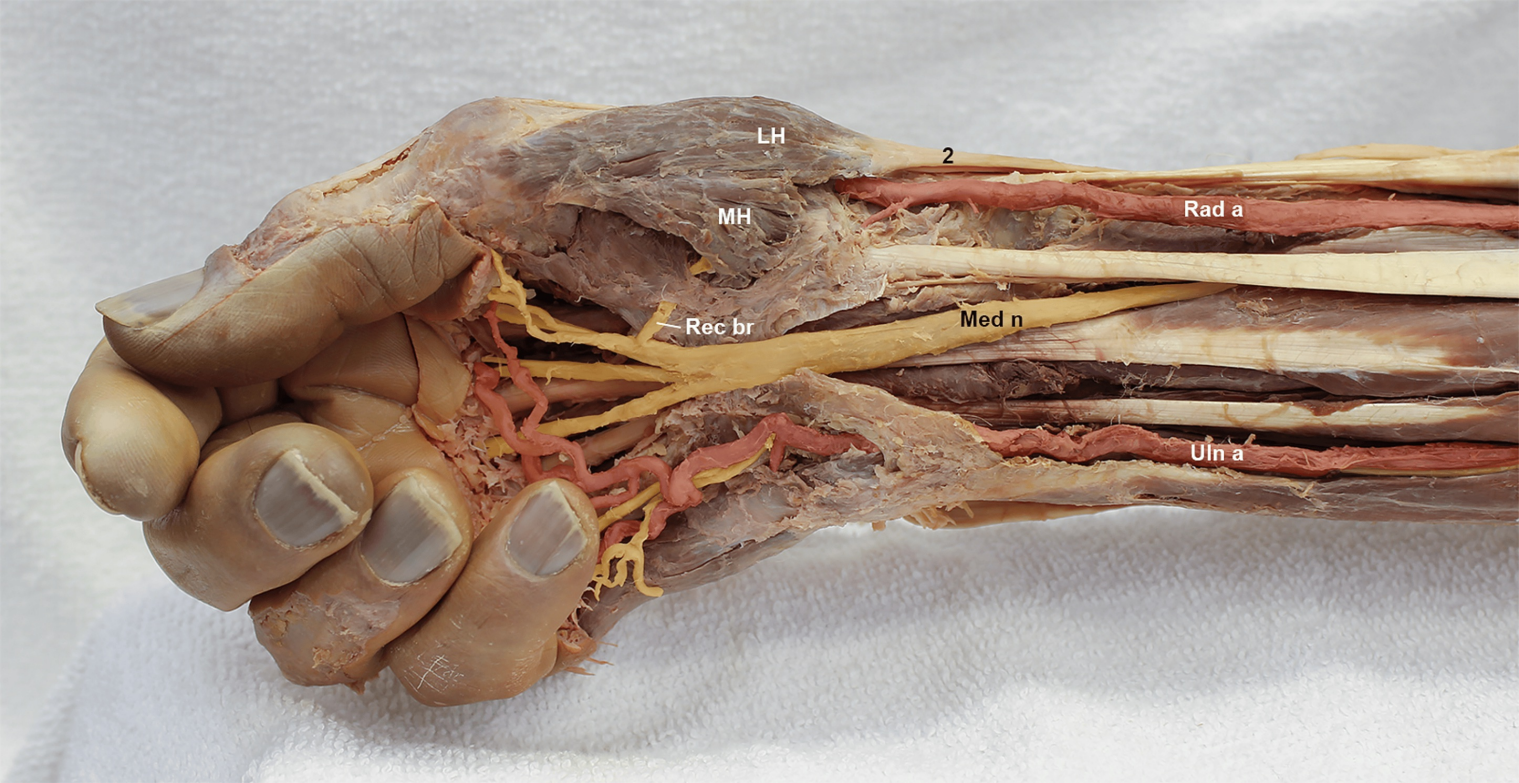
Anterior view of the right distal forearm and hand. The APL accessory tendon is attached to the lateral head of the APB.  Both APB heads are innervated by the recurrent branch of the median nerve. 2: accessory tendon of APL; LH: lateral head of APB; Med n: median nerve; MH: medial head of APB; Rad a: radial artery; Rec br: recurrent branch of the median nerve; Uln a: ulnar artery. Nerves are highlighted in yellow; arteries are highlighted in red.

The APB had two proximal attachments. The typical medial head originated from the flexor retinaculum, and an accessory lateral head connected with the APL, as described above (Figure 3). The APB had a typical distal insertion at the base of the proximal phalanx and extensor expansion of the thumb. The APB was innervated by the recurrent branch of the median nerve and received blood supply from the superficial palmar branch of the radial artery.

The EPB was absent bilaterally (Figure 1). No identifiable muscle belly was present, and no extensor tendon was inserted at the base of the proximal first phalanx.

## Discussion

In this case study, we characterize bilateral variations in thumb musculature observed in an anatomical donor. Specifically, each upper limb had an APL with two distal tendons, one that exhibited the typical attachment and one that connected to an accessory lateral head of APB, and each limb lacked an EPB. Each muscle retained its typical innervation, with the APL innervated by the posterior interosseous nerve and both heads of APB innervated by the recurrent branch of the median nerve.

Similar cases

Anatomic variations involving APL, APB, and EPB have been reported previously. Several studies have characterized supernumerary tendons of APL or EPB [4-6], absence of EPB [4-6], and additional origins of APB from the tendons of APL or palmaris longus [7-9]. A meta-analysis of variations in the first dorsal compartment (n= 2,478) reports the absence of EPB at 1.57% [10]. Digastric connections between the APL region of the extensor forearm and the thenar compartment are infrequently reported, and their prevalence has not been quantified. Case reports describe variable morphology of these digastric muscles, with some originating from a supernumerary proximal muscle belly [8,9,11] or having a distal muscle belly that is distinct from the APB [11,12]. Another case described unilateral fusion of APL and EPB, giving rise to multiple splitting tendons, one of which inserted into APB [13]. A case similar to ours involved bilateral APL accessory tendons that each formed the origin of the lateral portion of a two-headed APB; a tendinous slip connected the accessory APL to the trapezium [14]. Notably, all of these case reports of a digastric muscle associated with APL and/or APB had a normal EPB. All but two of the variations [11,14] were present unilaterally. This suggests that the bilateral, digastric APL-APB with absent EPB observed in the current study is relatively rare.

Embryonic development

This array of variations likely resulted from aberrant embryonic muscle patterning. During embryonic development, APL and EPB begin as a single muscle mass [1,3], and APL has 3 tendon slips: a dorsal slip to the first metacarpal, a middle slip to the trapezium, and a palmar slip to the opponens pollicis [15]. Subsequently, with typical development, the APL and EPB separate into distinct muscles, and the APL's tendinous slips to the trapezium and opponens pollicis regress. In the donor characterized in this case study, APL and EPB may have failed to separate, and EPB’s distal attachment may have failed to form, leading to the absence of EPB. The APL tendon slips to the opponens pollicis may have failed to regress, instead becoming associated with the APB. Developmental muscular anomalies like these could result from atypical signaling from the local mesenchyme during embryonic development [16].

Functional implications

This donor's anatomic variations may have altered the function of the thumb. APB’s accessory head and interconnection with the accessory tendon of APL could have shifted the muscle's force vector and diminished its stability, potentially altering the range of motion of the carpometacarpal and metacarpophalangeal joints. Thumb opposition may also have been weakened since this movement requires thumb abduction. The linear continuity of APL with APB might have increased the load on the APL muscle belly and tendon. However, the presence of one typical APL tendinous insertion on the base of the first metacarpal and the retention of APL's independent innervation suggest that this muscle's function was largely preserved. Finally, the absence of EPB would likely have weakened thumb extension and increased the strain on the extensor pollicis longus. Overall, this constellation of anomalies raises implications for decreasing dexterity in fine motor tasks and increasing strain during prolonged thumb abduction and extension.

Clinical implications

Anatomic variations like those described in this report raise potential implications for musculoskeletal conditions of the wrist. Such variations may influence susceptibility to de Quervain tenosynovitis (DQT), in which the tendons of APL and EPB become entrapped within the first dorsal compartment of the wrist [17]. While some studies have reported that DQT patients exhibit an elevated proportion of supernumerary APL tendon slips [18], others have found fewer APL tendon slips in DQT patients [19,20], illustrating the complex and nuanced function of these structures. Similarly, while the absence of EPB may be predicted to reduce susceptibility to DQT by leaving additional space within the first extensor compartment, it might instead elevate susceptibility by increasing the strain on APL, potentially leading to thickening or degeneration of the tendon sheath. Variations in APL and EPB could also influence susceptibility to intersection syndrome, which is characterized by stenosing tenosynovitis due to friction where the tendons of the first extensor compartment (APL and EPB) cross over those of the second extensor compartment (extensor carpi radialis longus and extensor carpi radialis brevis) [21]. If abnormal tendon interactions increase friction or pressure at the interface between the first two extensor compartments, the likelihood of intersection syndrome could increase. While structural variations in muscles of the first dorsal compartment are considered potentially contributory factors, they are not necessarily determinants for the development of inflammatory conditions. 

Supernumerary APL tendons may also present a beneficial resource for surgeries of the hand or wrist, as they may provide a convenient substrate for tendon transfer or graft procedures. Specifically, APL tendon slips have been transferred to replace the first dorsal interosseous [22] and extensor pollicis longus [23], and they have been used for grafts in flexor, extensor, and pulley reconstruction [24]. Thus, patients should be evaluated for the presence of additional APL tendons when planning such interventions.

## Conclusions

We describe a unique array of bilaterally symmetrical anatomic variations, in which an accessory APL tendon attached to a lateral accessory head of APB and EPB was absent, and each muscle retained its typical innervation. The unusual compartmental architecture might serve as a contributing factor for the development of conditions like de Quervain tenosynovitis and intersection syndrome, although these clinical associations remain inferential. This report primarily expands the understanding of surgically relevant anatomical variations affecting muscles of the first dorsal and thenar compartments.
